# Pregnant women’s decision-making capacity and adherence to iron supplementation in sub-Saharan Africa: a multi-country analysis of 25 countries

**DOI:** 10.1186/s12884-021-04258-7

**Published:** 2021-12-13

**Authors:** Betregiorgis Zegeye, Nicholas Kofi Adjei, Comfort Z. Olorunsaiye, Bright Opoku Ahinkorah, Edward Kwabena Ameyaw, Abdul-Aziz Seidu, Sanni Yaya

**Affiliations:** 1 HaSET Maternal and Child Health Research Program, Shewarobit Field Office, Shewarobit, Ethiopia; 2 Department of Public Health, Policy and Systems, University of Liverpool, Liverpool, UK; 3 Department of Public Health, Arcadia University, Glenside, PA USA; 4 School of Public Health, Faculty of Health, University of Technology Sydney, Ultimo, Australia; 5 Department of Population and Health, College of Humanities and Legal Studies, University of Cape Coast, Cape Coast, Ghana; 6 College of Public Health, Medical and Veterinary Sciences, James Cook University, Townsville, Queensland Australia; 7 School of International Development and Global Studies, Faculty of Social Sciences, University of Ottawa, 120 University Private, Ottawa, ON K1N 6N5 Canada; 8 The George Institute for Global Health, Imperial College London, London, UK

**Keywords:** Women autonomy, iron adherence, sub-Sahara Africa, DHS, Global health

## Abstract

**Background:**

Anaemia and related complications during pregnancy is a global problem but more prevalent in sub-Sahara Africa (SSA). Women’s decision-making power has significantly been linked with maternal health service utilization but there is inadequate evidence about adherence to iron supplementation. This study therefore assessed the association between household decision-making power and iron supplementation adherence among pregnant married women in 25 sub-Saharan African countries.

**Methods:**

We used data from the Demographic and Health Surveys (DHS) of 25 sub-Saharan African countries conducted between 2010 and 2019. Women's decision-making power was measured by three parameters; own health care, making large household purchases and visits to her family or relatives. The association between women’s decision-making power and iron supplementation adherence was assessed using logistic regressions, adjusting for confounders. The results were presented as adjusted odds ratio (AOR) with 95% confidence intervals (CIs).

**Results:**

Approximately 65.4% of pregnant married women had made decisions either alone or with husband in all three decisions making parameters (i.e., own health care, making large household purchases, visits to her family or relatives). The rate of adherence to iron medication during pregnancy was 51.7% (95% CI; 48.5–54.9%). Adherence to iron supplementation was found to be higher among pregnant married women who had decision-making power (AOR = 1.46, 95% CI; 1.16–1.83), secondary education (AOR = 1.45, 95% CI; 1.05–2.00) and antenatal care visit (AOR = 2.77, 95% CI; 2.19–3.51). Wealth quintiles and religion were significantly associated with adherence to iron supplementation.

**Conclusions:**

Adherence to iron supplementation is high among pregnant women in SSA. Decision making power, educational status and antenatal care visit were found to be significantly associated with adherence to these supplements. These findings highlight that there is a need to design interventions that enhance women’s decision-making capacities, and empowering them through education to improve the coverage of antenatal iron supplementation.

## Background

Anaemia is a major public health problem [1, 2], and it is estimated that more than two billion people are affected globally [1–3]. At least half of all anaemia can be attributed to iron deficiency [4], and the estimated prevalence is higher among women of reproductive age (15–49 years) [5]. Approximately 40% of pregnant women are anaemic worldwide [4]. There are, however, regional variations with the prevalence being higher in Africa (62.3%) and South-East Asia (53.8%) [6, 7].

The risks of anaemia and related complications have been shown to be high among pregnant women [4] because they need additional iron and folic acid to meet their nutritional needs and the growth of the fetus [4]. Anaemia related complications can contribute to miscarriage, intrauterine fetal death, preterm delivery, low birth weight and perinatal mortality [8, 9]. Iron and folic acid deficiency is usually the common cause of anaemia and related complications [4]. The World Health Organization (WHO) recommends daily oral iron and folic acid supplementation with 30 mg to 60 mg of elemental iron and 0.4 mg folic acid for pregnant women to prevent maternal anaemia, puerperal sepsis, low birth weight and preterm birth [4, 10].

Although there has been about 50% reduction in anaemia among reproductive-age women [11] as a result of the Sustainable Development Goals (SDGs) (Goal 2) [12] and the global nutrition targets of World Assembly for 2025 [11], global progress is still slow [13]. There is evidence of poor uptake of iron supplementation among pregnant women, particularly in SSA [14]. In a recent study conducted in 22 SSA countries, Ba and colleagues [14] showed that only 28.7% of pregnant women reported uptake of iron supplementation; even though estimates varied from lowest coverage (1.4%) in Burundi to highest (73%) in Senegal.

Studies in Malawi [15, 16], Ethiopia [17–19], and SSA [14] have identified several factors including socioeconomic condition, media exposure, geographic, and timing and number of antenatal care visits as factors linked with adherence to iron supplementation among pregnant women [14–19]. Furthermore, there is strong evidence that women’s autonomy is linked with maternal health services, including antenatal care, skilled delivery services and postnatal care [20–23].

However, to our knowledge, no study has assessed the relationship between women’s decision making and iron supplementation among married pregnant women in SSA countries. This present study, therefore, aimed to investigate the association between household decision-making power and iron adherence among married pregnant women in 25 SSA countries.

## Methods

### Data source

We used data from the Demographic and Health Surveys (DHSs) of 25 SSA countries conducted between 2010 and 2019. DHS is a nationally representative survey conducted across several low-and middle-income countries with financial and technical assistance from the United States Aid for International Development (USAID) and Inner-City Fund (ICF) International [24, 25]. DHS usually adopt a two-stage stratified sampling technique. In the first stage, clusters, also called enumeration area (EA) were selected using probability proportional to size. In the second stage, a fixed number of households (usually 25 to 30 households) were selected from clusters selected in stage one [26]. The countries were selected if the survey was conducted between 2010 and 2019, and outcome and explanatory variables were available. We included 120,131 married women in the final analysis from the individual recode (IR) file. We included only married women for the analysis because anaemia is common among married women [27]. Furthermore, the variables on decision making were only applicable to married women [28–30]. The DHS datasets are freely available for download at https://dhsprogram.com/data/available-datasets.cfm. We also followed the guidelines for Strengthening of Observational studies in Epidemiology (STROBE) [31]. Details about selected countries, year of survey and sample are shown in Table 1 below.

**Table 1:** Year of survey for each studied countries and sampled population

Country	Year of survey	Sampled population (Weighted)	Iron adherence (%)
Angola	2015/16	4344	51.71
Burkina Faso	2010	9288	55.14
Benin	2017/18	6923	70.27
Burundi	2016/17	3522	3.83
Congo Democratic Republic	2013/14	5141	10.95
Cote d’voire	2011/12	3427	39.16
Cameroon	2018/19	3914	67.01
Ethiopia	2016	3031	14.94
Gabon	2012	2373	70.31
Ghana	2014	3317	68.51
Gambia	2013	4835	52.81
Guinea	2018	4075	32.73
Kenya	2014	4003	16.76
Comoros	2012	1498	51.37
Liberia	2019/20	2963	92.99
Mali	2018	4575	53.77
Malawi	2016/17	10,026	39.18
Rwanda	2014/15	3842	5.48
Sierra-Leone	2019	5818	52.74
Senegal	2010/11	6976	76.65
Chad	2014/15	5195	31.04
Togo	2013/14	3951	45.16
Uganda	2016	7512	27.38
Zambia	2018/19	5339	83.75
Zimbabwe	2015	3391	50.94
Total		119,279	

### Study variables

#### Outcome variable

The outcome variable was iron supplement adherence. Information about the use of iron supplement was obtained from women who had a live birth within 5 years preceding the survey, by asking whether she took iron tablets or syrup for 90 days and above during pregnancy of last birth. We re-coded responses into a binary variable (0 = No; 1 = Yes) as done in previous studies [14, 15, 32, 33].

#### Explanatory variables

The key explanatory variable of interest was women’s decision-making power. In the DHS, married women aged 15–49 years were asked three questions about decision making. Questions about who decides on “own (respondent’s) health”, “large household purchases”, and “families or relatives visits” were used to measure women’s decision-making power [28–30]. These variables were also used to indirectly assess whether or not a woman was empowered [28–30]. The variables were recoded into binary variables. Women who made decisions alone or together with husbands on all three aforementioned decision-making parameters were coded as “1” while those whose responses were not in the affirmative were categorized as otherwise and coded as “0” [28].

Other explanatory variables included women’s age [15–49], women’s educational status (no formal education, primary school, secondary school, higher), husband’s educational status (no formal education, primary school, secondary school, higher), occupation (not working, professional/technical/managerial, agricultural, manual, others), parity [1–3, 5], 5+), place of residence (urban, rural), religion (Christian, Muslim, others) and number of antenatal care (ANC) visit (< 4 visits, 4+ visits). We also included wealth index (poorest, poorer, middle, richer, richest). In the survey, wealth index was computed using durable goods, household characteristics and basic services [34]. Other variables included exposure to media (newspaper, radio or television (TV)) which was assessed in terms of frequency (no exposure “no” or less than once a week “yes”).

### Statistical analysis

First, descriptive statistics were performed to obtain the prevalence of iron adherence and it distribution across the outcome, explanatory variables. Second, we conducted bivariate logistic regression analysis with each of the explanatory variables and the outcome variable (iron adherence) to select candidate explanatory variables for the multivariable logistic regression model, only variables that were statistically significant (*P* ≤ 0.05) in the bivariate logistic regression analysis were included in the multivariable logistic regression. Third, a multicollinearity test was conducted using variance inflation factor (VIF) to check for collinearity among selected variables. The test showed no evidence of collinearity among the variables (Mean VIF = 2.06, Max VIF = 4.81, Min VIF = 1.07). Finally, we performed a multivariable logistic regression (MLR) to assess the association between the selected explanatory variables and outcome variable. The goodness-of-fit of the regression model was assessed using Hosmer-Lemeshow [35], and we observed better fitting model (*P* = 0.3071). The results were presented using adjusted odd ratio (AOR) at 95% confidence interval (CI). The analysis was carried out using Stata version-14 software (Stata Corp, College Station, Texas, USA). We used the “svyset” command in Stata to account for the complex survey design including weight, cluster and strata.

### Ethical considerations

We used publicly available secondary data for analysis of this study (available at: https://dhsprogram.com/data/available-datasets.cfm). Ethical procedures were conducted by institutions that funded, commissioned, and managed the surveys. Thus no further ethical clearance was required. All data were anonymized prior to the authors receiving the data. For further details related to ethical issues, see http://goo.gl/ny8T6X.

## Results

### Socio-demographic characteristics

A total of 119,279 pregnant married women were included in this study and their socio-demographic characteristics are shown in Table 1. Of them, about 7.9% were 15–19 years old. More than a quarter (27.5%) and one-fifth (21.1%) of the respondents and their husbands had no formal education respectively. About one in four (25.3%) of the respondents had no job and 35.3% were living in rural areas.

### Distribution of iron supplementation adherence across explanatory variables

The prevalence of iron supplementation adherence by explanatory variables is shown in Table 2. We observed that the prevalence of iron supplementation adherence varied across socio-demographic sub-groups; for example, adherence to iron supplementation was found to be higher among respondents with higher education (65.4%) compared to those with no education (39.6%). Iron supplementation adherence varied approximately from 23.6 to 52.6% among Muslim and Christian married women respectively. Higher prevalence of iron supplementation was also observed among married women with 4 and above ANC visit (59.2%) compared to those with less than 4 ANC visits (29.9%).

**Table 2:** Prevalence of iron supplementation adherence among married pregnant women by explanatory variables. Evidence from 25 SSA countries DHSs

Variables	Numbers (Weighted %)	Iron adherence (Weighted %)		Chi-square, ***P***-Value
		No	Yes	
Overall prevalence	120,131 (51.7%)			
**Decision making**				χ2 = 55.89, *P* < 0.001
No	127,476 (34.59)	56.04	43.96	
Yes	90,356 (65.41)	44.14	55.86	
**Age in years**				χ2 = 54.03, *P* < 0.001
15–19	15,381 (7.86)	56.62	43.38	
20–24	40,182 (19.88)	55.31	44.69	
25–29	49,269 (21.60)	47.89	52.11	
30–34	42,258 (16.88)	43.70	56.3	
35–39	35,798 (14.56)	44.99	55.01	
40–44	25,700 (11.73)	39.05	60.95	
45–49	19,483 (7.51)	42.25	57.75	
**Women’s educational status**				χ2 = 98.12, *P* < 0.001
No formal education	99,491 (27.46)	60.40	39.60	
Primary school	75,431 (38.90)	50.23	49.77	
Secondary school	45,666 (29.65)	41.07	58.93	
Higher	7473 (3.98)	34.62	65.38	
**Husband’s educational status**				χ2 = 66.06, *P* < 0.001
No formal education	86,384 (21.13)	53.76	46.24	
Primary school	57,989 (26.62)	54.87	45.13	
Secondary school	57,884 (44.91)	46.05	53.95	
Higher	14,720 (7.34)	32.97	67.03	
**Women occupation**				χ2 = 96.77, *P* < 0.001
Not working	58,510 (25.30)	12.79	14.88	
Professional/technical/managerial	7840 (5.41)	1.92	3.28	
Agricultural	79,991 (29.67)	13.86	8.64	
Manual	15,708 (3.39)	1.65	1.91	
Others	55,827 (36.23)	18.08	23.00	
**Wealth index**				χ2 = 221.49, *P* < 0.001
Poorest	51,782 (17.92)	6.53	4.66	
Poorer	47,375 (20.66)	12.18	6.43	
Middle	45,144 (20.72)	12.11	11.67	
Richer	42,578 (20.59)	9.99	14.19	
Richest	41,192 (20.11)	7.48	14.75	
**Reading newspaper**				χ2 = 48.72, *P* < 0.001
No	196,334 (81.04)	51.09	48.91	
Yes	32,163 (18.96)	38.27	61.73	
**Listening radio**				χ2 = 32.37, *P* < 0.001
No	98,580 (45.55)	53.51	46.49	
Yes	129,986 (54.45)	44.73	55.27	
**Watching television**				χ2 = 88.02, *P* < 0.001
No	146,839 (38.71)	59.00	41.00	
Yes	81,567 (61.29)	43.57	56.43	
**Parity**				χ2 = 12.86, *P* = 0.0769
1–2	69,399 (30.72)	52.15	47.85	
3–4	63,805 (30.02)	45.96	54.04	
5+	79,947 (39.26)	46.81	53.19	
**Place of residence**				χ2 = 106.61, *P* < 0.001
Urban	72,778 (64.71)	43.66	56.34	
Rural	155,293 (35.29)	61.51	38.49	
**Religion**				χ2 = 24.20, *P* < 0.001
Christian	133,400 (93.53)	47.41	52.59	
Muslim	84,097 (0.36)	76.65	23.35	
Others	10,987 (6.11)	63.01	36.99	
**Number of ANC visit**				χ2 = 262.36, *P* < 0.001
< 4	65,290 (24.89)	70.06	29.94	
> = 4	79,314 (75.11)	40.77	59.23	

### Prevalence of iron supplementation adherence across countries

**Fig. 1:**
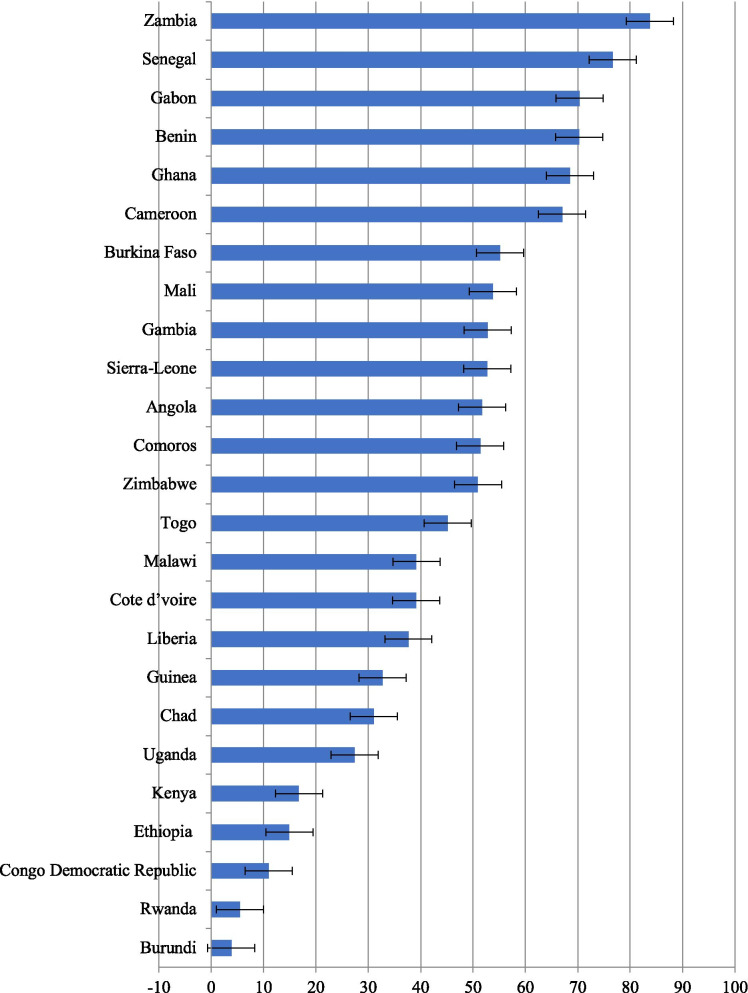
Prevalence of iron adherence among married women: Evidence from 25 SSA countries DHSs

The prevalence of iron supplementation adherence across 25 SSA countries is shown in Fig. 1. We observed the lowest prevalence of iron adherence in Burundi (3.8%), Rwanda (5.5%), Congo Democratic Republic (11%), Ethiopia (14.9%) and Kenya (16.8%). The highest prevalence of iron adherence was observed in Zambia (83.8%) followed by Senegal (76.7%), Gabon (70.3%), Benin (70.3%), Ghana (68.5%) and Cameroon (67%).

### Association between women’s decision-making power and iron supplementation adherence

#### Bivariate logistic regression results

Table 3 shows results of the bivariate and multivariable logistic regressions. The bivariate analysis showed that women’s decision-making power was significantly associated with adherence to iron supplementation. We also found women’s age, women’s educational status, husband’s educational status, women’s occupation, wealth index, media exposure, parity, place of residence, religion and number of ANC visit to be significantly associated with adherence to iron supplementation among married women in SSA.

**Table 3:** Bivariate and multivariable logistic regression output for women decision making power and iron adherence among married women: Evidence from 25 SSA countries DHSs

Variables	Model I COR [95% CI]	***P***-value	Model II AOR [95% CI]	***P***-value
**Decision making**				
No	Ref		Ref	
Yes	1.61 (1.29–2.01)	**< 0.001**	1.46 (1.16–1.83)	**0.001**
**Age in years**				
15–19	Ref		Ref	
20–24	1.05 (0.71–1.55)	0.788	0.84 (0.55–1.28)	0.427
25–29	1.42 (0.97–2.07)	0.069	0.94 (0.61–1.45)	0.793
30–34	1.68 (1.08–2.59)	**0.019**	1.10 (0.67–1.80)	0.692
35–39	1.59 (1.05–2.41)	**0.027**	1.02 (0.63–1.63)	0.923
40–44	2.03 (1.29–3.20)	**0.002**	1.43 (0.86–2.38)	0.167
45–49	1.78 (0.94–3.37)	0.075	1.59 (0.80–3.15)	0.178
**Women’s educational status**				
No formal education	Ref		Ref	
Primary school	1.51 (1.18–1.92)	**0.001**	1.30 (1.00–1.70)	**0.047**
Secondary school	2.18 (1.69–2.82)	**< 0.001**	1.45 (1.05–2.00)	**0.021**
Higher	2.88 (1.67–4.95)	**< 0.001**	1.36 (0.65–2.82)	0.405
**Husband’s educational status**				
No formal education	Ref		Ref	
Primary school	0.95 (0.71–1.27)	0.758	0.92 (0.68–1.23)	0.584
Secondary school	1.36 (1.06–1.73)	**0.014**	0.82 (0.63–1.07)	0.163
Higher	2.36 (1.58–3.52)	**< 0.001**	0.98 (0.61–1.57)	0.953
**Women occupation**				
Not working	Ref		Ref	
Professional/technical/managerial	1.47 (0.94–2.28)	0.083	0.94 (0.56–1.55)	0.813
Agricultural	0.53 (0.40–0.70)	**< 0.001**	0.92 (0.68–1.25)	0.615
Manual	0.99 (0.60–1.64)	0.984	0.82 (0.48–1.39)	0.468
Others	1.09 (0.86–1.37)	0.450	0.91 (0.71–1.15)	0.445
**Wealth index**				
Poorest	Ref		Ref	
Poorer	0.73 (0.52–1.03)	0.075	0.68 (0.46–0.99)	**0.049**
Middle	1.34 (0.94–1.92)	0.101	1.11 (0.65–1.91)	0.683
Richer	1.98 (1.34–2.94)	**0.001**	1.35 (0.75–2.44)	0.312
Richest	2.76 (1.84–4.13)	**< 0.001**	1.55 (0.82–2.94)	0.173
**Reading newspaper**				
No	Ref		Ref	
Yes	1.68 (1.30–2.18)	**< 0.001**	1.08 (0.79–1.47)	0.622
**Listening radio**				
No	Ref		Ref	
Yes	1.42 (1.20–1.67)	**< 0.001**	0.99 (0.81–1.22)	0.981
**Watching television**				
No	Ref		Ref	
Yes	1.86 (1.52–2.27)	**< 0.001**	0.94 (0.75–1.18)	0.614
**Parity**				
1–2	Ref		Ref	
3–4	1.28 (1.01–1.61)	**0.034**	1.29 (0.98–1.69)	0.062
5+	1.23 (0.95–1.59)	0.100	1.25 (0.89–1.76)	0.183
**Place of residence**				
Urban	Ref		Ref	
Rural	0.48 (0.37–0.62)	**< 0.001**	0.85 (0.53–1.34)	0.492
**Religion**				
Christian	Ref		Ref	
Muslim	0.27 (0.06–1.23)	0.092	0.20 (0.05–0.76)	**0.018**
Others	0.52 (0.35–0.79)	**0.002**	0.74 (0.48–1.14)	0.178
**Number of ANC visit**				
< 4	Ref		Ref	
> = 4	3.40 (2.69–4.29)	**< 0.001**	2.77 (2.19–3.51)	**< 0.001**

Notes: Ref reference

#### Multivariable logistic regression results

As shown in Table 3, we found a significant association between women’s decision making power and adherence to iron supplementation, where the odds of adherence was seen to be higher among married women who had decision-making power on all the decision making parameters (AOR = 1.46, 95% CI; 1.16–1.83) compared to married women who had no decision-making power. Furthermore, we observed a higher probability of iron supplementation adherence for married women who had secondary education (AOR = 1.45, 95% CI; 1.05–2.00) compared to married women who had no formal education. Higher odds of iron adherence was also observed among married women who had four or more ANC visit (AOR = 2.77, 95% CI; 2.19–3.51) compared to those who had less than four ANC visits. The likelihood of adherence to iron supplementation was found to lower among poorer households (AOR = 0.68, 95% CI; 0.46–0.99) and Muslim women (AOR = 0.20, 95% CI; 0.05–0.76).

Regarding country specific findings, married women who had decision making power were more likely to have adherence to iron supplementation in Angola (AOR = 1.36, 95% CI; 1.19–1.55), Cameroon (AOR = 1.39, 95% CI; 1.19–1.63), Gambia (AOR = 1.30, 95% CI; 1.15–1.47), Liberia (AOR = 1.79, 95% CI; 1.23–2.62), Malawi (AOR = 1.13, 95% CI; 1.04–1.23), Senegal (AOR = 1.54, 95% CI; 1.28–1.84), Uganda (AOR = 1.49, 95% CI; 1.34–1.66) and Zambia (AOR = 1.21, 95% CI; 1.04–1.42). Surprisingly, the inverse was found in Mali (AOR = 0.70, 95% CI; 0.57–0.86) and Togo (AOR = 0.74, 95% CI; 0.63–0.87) (Table 4).

**Table 4:** Bivariate and multivariable logistic regression output for women decision making power and iron adherence among married women by country: Evidence from 25 SSA countries DHSs

Countries	Model I COR[95% CI]	***P***-value	Model II AOR[95% CI]	***P***-value
Angola	1.44 (1.27–1.63)	**< 0.001**	1.36 (1.19–1.55)	**< 0.001**
Burkina Faso	1.21 (1.06–1.38)	**0.004**	1.15 (1.00–1.33)	0.042
Benin	1.12 (1.00–1.25)	**0.033**	0.94 (0.84–1.06)	0.370
Burundi	1.01 (0.71–1.44)	0.932	1.02 (0.71–1.47)	0.883
Congo Democratic Republic	1.15 (0.96–1.38)	0.128	1.06 (0.87–1.28)	0.551
Cote d’Ivoire	1.12 (0.95–1.33)	0.162	0.91 (0.75–1.10)	0.371
Cameroon	1.90 (1.65–2.18)	**< 0.001**	1.39 (1.19–1.63)	**< 0.001**
Ethiopia	1.19 (0.96–1.47)	0.098	1.05 (0.84–1.32)	0.628
Gabon	1.48 (1.25–1.76)	**< 0.001**	0.86 (0.60–1.23)	0.435
Ghana	1.06 (0.92–1.23)	0.370	1.08 (0.92–1.27)	0.324
Gambia	1.33 (1.18–1.50)	**< 0.001**	1.30 (1.15–1.47)	**< 0.001**
Guinea	1.00 (0.87–1.16)	0.892	0.92 (0.79–1.08)	0.346
Kenya	1.04 (0.88–1.24)	0.598	0.96 (0.80–1.15)	0.680
Comoros	1.23 (0.99–1.52)	0.052	1.19 (0.95–1.50)	0.121
Liberia	1.85 (1.36–2.51)	**< 0.001**	1.79 (1.23–2.62)	**0.002**
Mali	0.87 (0.72–1.05)	0.175	0.70 (0.57–0.86)	**0.001**
Malawi	1.22 (1.13–1.32)	**< 0.001**	1.13 (1.04–1.23)	**0.003**
Rwanda	0.95 (0.71–1.26)	0.727	0.93 (0.69–1.25)	0.633
Sierra-Leone	1.20 (1.07–1.34)	**0.001**	1.11 (0.98–1.24)	0.076
Senegal	1.62 (1.37–1.93)	**< 0.001**	1.54 (1.28–1.84)	**< 0.001**
Chad	1.00 (0.86–1.17)	0.906	1.02 (0.86–1.20)	0.786
Togo	0.75 (0.65–0.87)	**< 0.001**	0.74 (0.63–0.87)	**< 0.001**
Uganda	1.54 (1.39–1.71)	**< 0.001**	1.49 (1.34–1.66)	**< 0.001**
Zambia	1.30 (1.12–1.50)	**< 0.001**	1.21 (1.04–1.42)	**0.013**
Zimbabwe	0.92 (0.79–1.07)	0.287	0.97 (0.82–1.14)	0.761

## Discussion

Using nationally representative data, we assessed the association between household decision- making power and adherence to iron supplementation among married women in 25 SSA countries. Overall, the results revealed that about 51.7% of married pregnant women in the selected countries reported intake of iron tablets/syrups for 90 days or more. This estimate is lower than what was found in previous SSA countries, where about 28.7% of women adhered to an intake of iron supplements [14]. These inconsistent findings may be attributed to the study population [14]. In this current study, only married pregnant woman were included in the analysis as opposed to unmarried women in prior studies [14]. We, however, observed variations in the prevalence of iron supplement adherence across SSA countries, with the lowest prevalence in Burundi (3.8%) and the highest prevalence in Zambia (83.8%).

We found women’s decision-making power to be positively associated with adherence to iron supplementation among pregnant married women. Although no known study assessed the relationship between women’s decision-making power and iron adherence in SSA, there is some evidence that women’s decision-making power is a contributing factor for better utilization of maternal health in some countries including Nepal [20], Bangladesh [36], India [21], Cameroon [23], Ethiopia [37] and Benin [38]. Spousal communication has been shown to be vital for women’s decision-making power [36], where prior studies suggest that poor communication and non-support from partners may lead to poor uptake of maternal health services [36, 39]. Other possible explanations include socioeconomic status [40, 41] and cultural norm [42]. In a recent study conducted in Senegal Sougou et al. showed that women with higher socioeconomic status had better decision-making capacities [43].

Consistent with prior studies in Malawi [15], Ethiopia [17, 18] and SSA [14] we found that women who had formal education were more likely to use iron supplements than non-educated women. This is because educated women may be well informed about their health [14, 15, 44], have access to nutritional information [45] and may know the benefits of iron supplementation [46] Furthermore, they may be knowledgeable about maternal health services, which can enable them seek healthcare services [47, 48].

We found religion to be significantly associated with to adherence to iron supplementation. The likelihood of iron supplement intake was lower among Muslim than Christians. Although there is no prior evidence of the relationship between iron supplement intake and religion, a study conducted in Nigeria showed no significant difference in the uptake of maternal health services between Muslim and Christian women [49]. However, studies in Ghana [50], Nigeria [51, 52] and Benin [53] suggest a significant association between religion and maternal healthcare access and service utilization. 

Finally, a significant association was found between the number of ANC visits and iron supplement adherence. Women who had four or more visits were more likely to use iron supplements than those who had less than four ANC visits, consistent with prior studies [14, 16, 19, 54]. Pregnant women generally receive iron supplementation through ANC visits at health facilities [10]; thus, this finding is expected as health facilities may find ANC visits as a good opportunity for the distribution of iron supplements for pregnant women [10, 11].

### Strength and limitation of the study

The major strengths of our study include the large nationally representative sample and a multi-country analysis. Nonetheless, some limitations were also observed. First, a causal-effect relationship cannot be established because of the cross-sectional nature of the study. Second, the DHS relied on self-reported data which may be prone to recall bias. Lastly, due to data availability and constrains, we used surveys that were conducted at different time points in the selected countries.

## Conclusion

This study shows that approximately 65.4% of married pregnant women had decision-making power, and about half (51.7%) used iron supplements during pregnancy. Pregnant women with decision making power were more likely to use iron supplements. Socio-demographic factors including women’s educational level, household economic status, religion and number of ANC visits were significantly associated with adherence to iron supplementation. These findings highlight that there is a need to design interventions that enhance women’s decision-making capacities, and empowering them through education to improve the coverage of antenatal iron supplementation.

## Data Availability

Data for this study were sourced from Demographic and Health surveys (DHS) and available here: http://dhsprogram.com/data/available-datasets.cfm.
